# Unexpected effects of treating insulin-resistant obese women with high-dose D-chiro-inositol: opening Pandora’s box

**DOI:** 10.3389/fendo.2025.1399308

**Published:** 2025-04-01

**Authors:** Sabrina Basciani, Maurizio Nordio, Maria Letizia Spizzichini, Lucio Gnessi

**Affiliations:** ^1^ Section of Medical Pathophysiology, Food Science and Endocrinology, Department of Experimental Medicine, Sapienza University of Rome, Rome, Italy; ^2^ Department of Experimental Medicine, Sapienza University of Rome, Rome, Italy; ^3^ The Experts Group on Inositol in Basic and Clinical Research, and on PCOS (EGOI-PCOS), Rome, Italy

**Keywords:** D-chiro-inositol, insulin resistance, hyperandrogenism, lifestyle, menstrual regularity, obesity

## Abstract

**Purpose:**

The combination of lifestyle changes and nutraceuticals, such as inositols, can reduce excess weight, leading to a reduction in insulin resistance and a normalization of the metabolic profile. As such, this study investigated the metabolic and hormonal changes observed in women who were overweight/obese with insulin resistance undergoing a hypocaloric Mediterranean diet combined with high-dose D-chiro-inositol (DCI) treatment.

**Methods:**

In total, 48 insulin-resistant women between 25 and 40 years old, with a body mass index (BMI) between 26 and 32 were divided into two groups: both groups followed a hypocaloric Mediterranean diet for 4 months, and patients in the treated group also underwent treatment with 2400 mg/day of DCI for the same period. We evaluated the homeostasis model assessment (HOMA) index, body weight, BMI, blood glucose, fasting insulin, lipid profile [cholesterol, low-density lipoprotein (LDL), high-density lipoprotein (HDL), and triglycerides] and hormonal profile [total testosterone, androstenedione, dehydroepiandrosterone sulfate (DHEAS), sex hormone-binding globulin (SHBG), estradiol, follicle-stimulating hormone (FSH), luteinizing hormone (LH), and menstrual length] at baseline and at the end of treatment.

**Results:**

After 4 months, both groups displayed a significant improvement in insulin sensitivity, as reflected by a reduction in the HOMA index, blood glucose level, fasting insulin level, and lipid profile. Furthermore, we observed a significant decrease in body weight and BMI in both groups. However, the evaluation of the hormonal profiles revealed unexpected findings, with the DCI-supplemented group exhibiting hyperandrogenism and menstrual irregularity, as demonstrated by the significant increase of total testosterone, androstenedione, LH, and menstrual length.

**Conclusion:**

The study strengthens the evidence regarding the metabolic benefits of the hypocaloric Mediterranean diet, independent from the association with DCI, on women with insulin resistance and excess weight, while also acknowledging the complex hormonal impact of high-dose DCI supplementation for medium-to-long periods.

## Introduction

Insulin resistance (IR) is a dysmetabolic condition resulting in a reduced response to insulin from peripheral target tissues. Therefore, tissues require higher concentrations of insulin to achieve a physiological response, leading to compensatory hyperinsulinemia, which is the typical diagnostic hallmark of IR (1).

The direct metabolic consequences of IR include hyperglycemia and obesity, which may lead to inflammation, hypertension, endothelial dysfunction, cardiovascular diseases, metabolic syndrome, non-alcoholic steatosis, and type 2 diabetes mellitus (2–5).

Aside from genetic susceptibility, IR and obesity typically stem from an unhealthy lifestyle, examples of which include a nutritional imbalance with weight gain and an excess buildup of adipose tissue, insufficient physical activity, increased sodium intake, and glucose toxicity and lipotoxicity from excessive circulating free fatty acids (6). In addition, several studies suggest the impairment of gut microbiota, as an environmental factor, may be related to the progression of IR, obesity, and metabolic disturbances (7, 8).

Therefore, lifestyle intervention is crucial for both the treatment and prevention of IR in at-risk patients, as compelling evidence has demonstrated that a moderate and well-tailored weight loss—by as little as 5%–10%—can lead to several health benefits, including a reduction of blood pressure and positive changes in insulin sensitivity and inflammatory biomarkers (9).

In this context, the hypocaloric regimen defined as the Mediterranean diet can improve IR in obese individuals when compared to other dietary approaches, especially in terms of insulin levels, homeostasis model assessment index for IR (HOMA-IR), and inflammation (10–12).

When lifestyle modifications alone are not sufficient to achieve clinically relevant weight loss and metabolic recovery, supplementation with natural compounds may be of great help, accelerating the recovery process and avoiding, or at least postponing, pharmacological interventions and/or surgical approaches.

Among natural molecules with insulin-sensitizing properties, inositols have proven effective in normalizing IR and hyperinsulinemia in dysmetabolic patients, mainly in overweight and obese women, or in women with polycystic ovary syndrome (PCOS) (13–17). D-chiro-inositol (DCI)—the second-most represented isomer of the inositol family—regulates insulin secretion, the mitochondrial respiratory chain, and glycogen storage. The first evidence of the importance of DCI in these processes derives from the observation of a higher DCI content in the body areas deputed to glycogen storage, mainly adipose cells, muscles, and the liver (18). As such, physicians use this molecule to treat insulin dysfunctions in a panel of conditions characterized by metabolic abnormalities (18). Moreover, some studies have suggested that a deficiency of DCI and its high urinary clearance correlate with IR and hyperinsulinemia, leading to the onset of diabetes and metabolic disorders, as observed in patients with PCOS (17, 19–22). There is plenty of clinical evidence on the effectiveness of DCI as an insulin sensitizer, and as a relevant molecule in the field of endocrinology and nutrition (16, 19, 22, 23).

On these premises, the present study aims to evaluate metabolic changes experienced by insulin-resistant overweight/obese women on a hypocaloric Mediterranean diet supplemented with DCI for a period of 4 months.

## Materials and methods

### Study design and participants

This was an open-label, controlled, interventional study carried out from June to October 2022 at the Section of Medical Pathophysiology, Food Science, and Endocrinology, Department of Experimental Medicine, Sapienza University of Rome. This study was registered on ClinicalTrials.Gov (Identifier: NCT05348941; Approval number: CE6422; Board name: Kemeso) and followed the Good Clinical Practice guidelines and the Declaration of Helsinki. Accordingly, all patients provided written informed consent.

In total, 48 Italian women aged between 25 and 40 years, with a body mass index (BMI) of between 26 and 32 and a diagnosis of IR (HOMA index ≥2.5), were enrolled in our department.

Exclusion criteria included (i) treatment with drugs or supplements that interfere with the mechanism of action of insulin and (ii) pregnancy and breastfeeding.

The sample size was calculated according to previous literature, which demonstrated a decrease in the HOMA index from 5.05 ± 1.51 at baseline to 3.05 ± 0.85 after a 3-month treatment with DCI (23). Setting a power of 90% and a chance of type-1 error equal to 0.05 and considering an adherence rate equal to approximately 50% (24), we obtained a sample size equal to 24 patients per arm, resulting in 48 patients in total.

The 48 enrolled women were divided into two groups based on availability and consent. The treated group (n= 24) received 2400 mg/day of DCI orally in addition to a hypocaloric Mediterranean diet, consisting of fruit, vegetables, olive oil, legumes, cereals, and fish, providing a fixed percentage of carbohydrates, fats (mainly monounsaturated), and proteins (55%, 25%, and 20%, respectively). The patients in the control group (n= 24) followed the same hypocaloric Mediterranean diet in the absence of supplementation. All patients were monitored for 4 months, with the possibility of a second follow-up after 2 more months of treatment.

### Measurement

The aim of this study was to evaluate the effects of a 4-month oral supplementation with a high dose of DCI (2400 mg/day) in association with a hypocaloric Mediterranean diet on IR.

DCI dosage was chosen according to the prior work by Nestler (25, 26).

The HOMA index was the primary outcome, while body weight, BMI, blood glucose levels, fasting insulin, lipid profile [cholesterol, low density lipoprotein (LDL), high density lipoprotein (HDL), and triglycerides], and hormonal profile [total testosterone, androstenedione, dehydroepiandrosterone sulfate (DHEAS), sex hormone-binding globulin (SHBG), estradiol, follicle-stimulating hormone (FSH), luteinizing hormone (LH), and menstrual length] represented secondary outcomes. Blood samples from all patients were collected by venipuncture during the follicular phase at baseline and after 4 months of diet and diet + DCI treatment.

After the blood samples were centrifuged at 1,000 × gravitational units (g) for 10 minutes to separate the serum, the serum was stored at − 20°C until assayed.

All the analyses were performed using commercial kits, at Policlinico Umberto I hospital (Rome, Italy). Insulin and glucose levels were assessed using a DPC Immulite 2000 analyzer (Euro/DPC, Llanberis, UK). Cholesterol was measured using an enzymatic cholesterol oxidase/peroxidase method (Beckman Coulter Diagnostics, Brea, CA, USA), and triglycerides were measured using an enzymatic assay (Beckman Coulter Diagnostics, Brea, CA, USA); HDL-cholesterol and LDL-cholesterol tests were performed (Beckman Coulter Diagnostics, Brea, CA, USA). Total testosterone and SHBG were measured using an ECLIA (Electrochemiluminescence immunoassay) kit (Roche Diagnostics, Mannheim, Germany); estradiol was measured using a competitive immunoassay (Access Immunoassay System, Estradiol, Beckman Coulter, Brea, CA, USA). DHEAS was measured using an enzymatic assay (Roche Diagnostics, Mannheim, Germany). Androstenedione was measured using an RIA kit (Beckman Coulter Diagnostics, Brea, CA, USA). FSH and LH were measured using ELISA kits. Free androgen index (FAI) was calculated as [(total testosterone/SHBG) x 100].

### Statistical analysis

Data were analyzed via Mann–Whitney U tests (2018 GraphPad Software 8.0.1, La Jolla, CA, USA); values are provided as median (25^th^ percentile – 75^th^ percentile). We considered a *p*-value of <0.05 to be statistically significant.

## Results

### General characteristics

All 48 patients completed the study. Baseline parameters and anthropometric measures were not significantly different between the control and treatment groups, as shown in **Table 1**.

**Table 1:** Anthropometric measurements at baseline in both groups.

	Control group	Treated group
Age (years)	33.5(28.5 – 36.75)	34.5(29.25 – 37.75)
Height (cm)	159(158 – 161)	160.5(159 – 164)
Body weight (kg)	70.95(68.68 – 72.55)	77.10*(71.15 – 80.15)
BMI	28.20(27.35 – 28.68)	29.55(27.38 – 31.10)
HOMA index	4.65(4.13 – 5.68)	4.60(3.30 – 5.33)
Menstrual length (days)	34(28 – 35.8)	28(28 – 29.8)

Values are expressed as median (25^th^ percentile – 75^th^ percentile). *p<0.05 vs the control group.BMI, body mass index; HOMA, homeostasis model assessment.

### HOMA index, blood glucose levels, fasting insulin

After 4 months, both groups demonstrated a significant improvement in the HOMA index, blood glucose levels, and fasting insulin levels.

In detail, the HOMA index in the treated group significantly decreased from 4.6 (3.3 – 5.33) to 3.25 (2.9 – 4.28), while in the control group, a significant reduction was also observed from 4.65 (4.13 – 5.65) to 3.35 (2.93 – 4.33).

The same pattern was recorded for both blood glucose and fasting insulin, as shown in **Table 2**. When analyzing the variations from baseline to 4 months between the two groups, no significant differences were found in the HOMA index or in blood glucose and fasting insulin levels.

**Table 2:** HOMA index, blood glucose, and fasting insulin in the control (diet alone) and treated (diet + DCI) groups at baseline (T0) and after 4 months (T4).

	Control group		Treated group	
	T0	T4	T0	T4
HOMA index	4.65(4.13 – 5.68)	3.35^***^(2.93 – 4.33)	4.60 (3.30 – 5.33)	3.25^***^(2.90 – 4.28)
Blood glucose (mg/dL)	111.00(107.25 – 117.00)	101.00^***^(98.00 – 110.75)	106.00(100.00 – 112.00)	102.50^*^(96.00 – 106.75)
Fasting insulin (mcU/dL)	17.40(15.58 – 19.50)	13.45^***^(11.88 – 16.38)	17.25(13.83 – 19.73)	13.00^***^(11.03 – 16.90)

Values are expressed as median (25^th^ percentile – 75^th^ percentile). *p<0.05 vs T0; ***p<0.001 vs T0.

### Body weight and BMI

The 4-month treatment significantly decreased body weight and BMI in both groups, as shown in **Table 3**. Likewise, after the study period there was no significant difference between the study group and the control.

**Table 3:** Body weight and BMI in the control (diet alone) and treated (diet + DCI) groups at baseline (T0) and after 4 months (T4).

	Control group		Treated group	
	T0	T4	T0	T4
Body weight (kg)	70.95(68.68 – 72.55)	67.00^***^(64.58– 71.30)	77.10(71.15 – 80.15)	73.40^***^(68.80 – 77.40)
BMI	28.20(27.35 – 28.68)	26.40^***^(25.83 – 27.68)	29.55(27.38 – 31.10)	28.20^***^(26.63 – 29.73)

Values are expressed as median (25^th^ percentile – 75^th^ percentile). ***p<0.001 vs T0.BMI, body mass index.

### Lipid profile

The 4-month treatment significantly ameliorated the lipid pattern in both groups, as shown in **Table 4**. In particular, we observed a significant reduction in cholesterol, LDL, and triglycerides and a significant increase in HDL levels. After the study period, there was no significant difference between the study group and the control.

**Table 4:** Cholesterol, LDL, HDL, and triglycerides in the control (diet alone) and treated (diet + DCI) groups at baseline (T0) and after 4 months (T4).

	Control group		Treated group	
	T0	T4	T0	T4
Cholesterol (mg/dL)	203.5(196 – 257.8)	187.5^*^(176.25 – 212.75)	214.5(188.75 – 243.5)	187.5^*^(174.25 – 200.75)
LDL (mg/dL)	143(116 – 160.25)	117.5^*^(102 – 138.75)	138.5(98.25 – 164.75)	119^*^(92.5 – 142.5)
HDL (mg/dL)	50.5(42 – 57)	56.5^*^(49.5 – 63.5)	48.5(43.25 – 57.75)	53^*^(46.75 – 59.75)
Triglycerides (mg/dL)	125.5(114.5 – 175.75)	115.5^*^(99.25 – 128.25)	139.5(106.5 – 76.25)	120^*^(99.25 – 132)

Values are expressed as median (25th percentile – 75th percentile). *p<0.05 vs T0.LDL, low-density lipoprotein cholesterol; HDL, high-density lipoprotein cholesterol.

### Hormonal profile

As the normalization of metabolic profile is often associated with an improvement in hormonal patterns and menstrual regularity, we also evaluated total testosterone, androstenedione, DHEAS, SHBG, FAI, estradiol, FSH, LH, and menstrual length as secondary outcomes.

**Figure 1:**
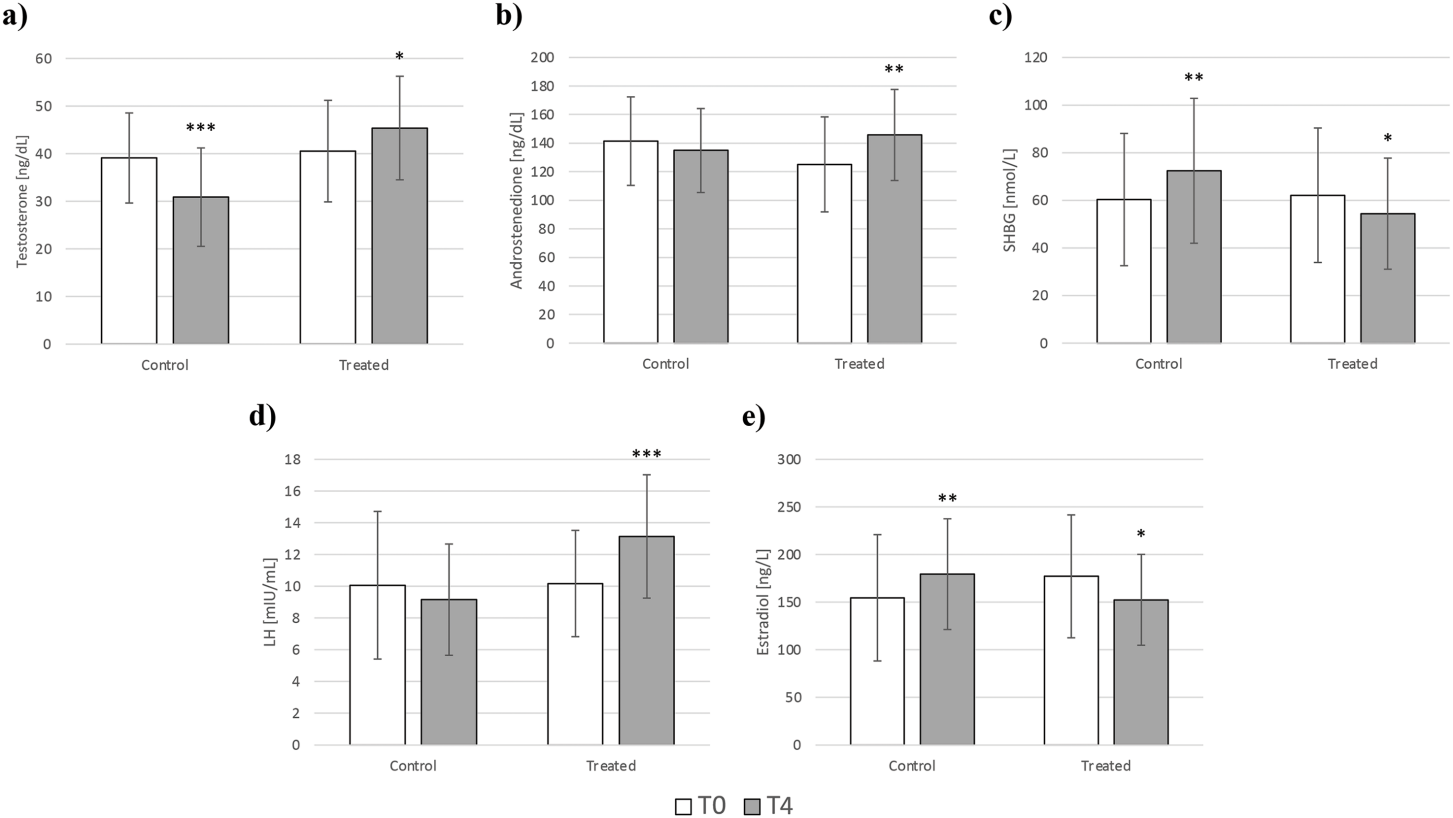
Changes in testosterone **(a)**, androstenedione **(b)**, SHBG **(c)**, LH **(d)**, and estradiol **(e)** in the control (diet alone) and treated (diet + DCI) groups at baseline (T0) and after 4 months (T4). Values are expressed as median (25^th^ percentile – 75^th^ percentile). *p<0.05 vs T0; **p<0.01 vs T0; ***p<0.001 vs T0. SHBG, sex hormone-binding globulin; LH, luteinizing hormone.

Total testosterone significantly decreased in the control group, while the opposite was observed in the study group. Indeed, the treated group experienced a significant increase in testosterone levels with respect to the baseline value (**Figure 1a**). Androstenedione remained unchanged in the control group but was also significantly increased in the treated group (**Figure 1b**).

SHBG was inversely affected when comparing the changes that occurred in the two groups. Indeed, the amount of SHBG significantly increased in the control group, while its levels significantly decreased in the group supplemented with high-dose DCI (**Figure 1c**).

Consequently, the FAI significantly decreased in the control group from 2.43 (1.70 – 3.44) to 1.56 (0.97 – 2.23) (*p*<0.01) while it significantly increased in the patients who received the supplementation, from 2.90 (1.64 – 4.02) to 3.06 (2.48 – 4.37) (*p*<0.01).

LH levels exhibited a contrary pattern to SHBG, increasing in the treated group with no change in the control group (**Figure 1d**).

Estradiol levels appeared to be higher after 4 months in the control group, increasing from 151.5 ng/L (95.5 – 202) to 189.5 ng/L (139 – 211.8) (*p*<0.01). In contrast, DCI supplementation contributed to a significant decrease in estradiol levels, from 179 ng/L (121.8 – 213.5) to 156 ng/L (124.8 – 175.8) (*p*<0.05) (**Figure 1e**). In both groups, no significant changes were observed for DHEAS and FSH. All the hormonal changes are shown in **Table 5**.

**Table 5:** Testosterone, androstenedione, SHBG, LH, and estradiol levels in the control (diet alone) and treated (diet + DCI) groups at baseline (T0) and after 4 months (T4).

	Control group		Treated group	
	T0	T4	T0	T4
Testosterone (ng/dL)	40.5(33.25 – 46)	28^***^(23.5 – 35.5)	42(32.75 – 48)	47.5^*^(39.5 – 49.75)
Androstenedione (ng/dL)	144.5(108.75 – 165.75)	131(112.5 – 150.25)	119(96.5 – 155.5)	148.5^**^(118 – 173.75)
SHBG (nmol/L)	58.5(34.75 – 83.25)	74^**^(45.75 – 97.5)	54.5(39.5 – 78.5)	49^*^(37.5 – 65.75)
FAI	2.43(1.70 – 3.44)	1.56^**^(0.97 – 2.23)	2.90(1.64 – 4.02)	3.06^***^(2.48 – 4.37)
LH (mIU/mL)	9.4(6.08 – 13.77)	8.65(6.43 – 11.53)	10(7.55 – 12.58)	12.95^***^(10.98 – 15.68)
Estradiol (ng/dL)	151.5(95.5 – 202)	189.5^**^(139 – 211.75)	179(121.75 – 213.5)	156^*^(124.75 – 175.75)
FSH (mIU/mL)	8.1(4.92 – 11.4)	8.05(5.98 – 11.4)	7.3(5.1 – 9.5)	8.15(6.1 – 11)
DHEAS (µg/dL)	127(88.5 – 191.5)	133.5(91.5 – 185.2)	112.5(93 – 171.5)	130(103.8 – 181.8)
Menstrual length (days)	34(28 – 35.75)	28.5^*^(28 – 32.8)	28(28 – 29.8)	32^***^(30 – 34)

Values are expressed as median (25^th^ percentile – 75^th^ percentile). *p<0.05 vs T0; **p<0.01 vs T0; ***p<0.001 vs T0.SHBG, sex hormone-binding globulin; FAI, free androgen index; LH, luteinizing hormone; FSH, follicle-stimulating hormone; DHEAS, dehydroepiandrosterone sulfate.

### Menstrual length

In agreement with the hormonal parameters, the menstrual cycle was also greatly affected by high-dose DCI. While the menstrual length in the control group was significantly improved from 34 (28 – 35.8) to 28.5 (28 – 32.8) days, patients who received DCI supplementation experienced a significant increase in menstrual length, from 28 (28 – 29.8) to 32 (30 – 34) days.

Due to these circumstances, namely the significant increase in androgen levels and the extension of menstrual cycle, the authors decided that the study needed to be discontinued for patient safety concerns. Thereby, a second follow-up after 2 further months of treatment, as previously scheduled, was no longer considered.

## Discussion

Obesity is a chronic condition that is associated with several metabolic and non-metabolic comorbidities, impacting life quality and expectancy and increasing public health costs worldwide (27–30). Among the drivers of excess weight and related complications, IR stands out as a primary contributing factor (31–33). Indeed, IR and prediabetes are on the increase worldwide, with alarming predictions for the next 15–20 years (34). Clearly, these data underscore the pressing need for lifestyle adjustments to tackle weight gain and associated disorders.

The recommended first-line approach for weight loss relies on adequate physical activity and healthy eating habits (35). In this context, the Mediterranean diet is associated with several benefits in terms of reducing IR, and lowering the risk of developing diabetes, metabolic syndrome, and cardiovascular diseases (36, 37).

Furthermore, nutraceutical interventions are currently employed on a large-scale basis as potential treatments for weight management and insulin sensitivity improvement (38, 39).

Consequently, inositols have attracted much attention, as several studies have confirmed their effectiveness in improving IR and excess weight-associated complications in patients with PCOS, metabolic syndrome, and diabetes (40).

Aside from increasing insulin sensitivity, inositols, and notably DCI, have been proven to be able to correct the imbalance between energy intake and energy output (41), leading to a reduction in oxidative stress (42), inflammation (43), and BMI (16, 44).

As such, this study intended to examine the benefits, in terms of metabolic and hormonal rebalance, of supplementing insulin-resistant overweight/obese women with DCI in combination with a hypocaloric Mediterranean diet.

In agreement with the results of the Nestler research group (25, 26), we opted for a treatment with high-dose DCI (45), as prior data has demonstrated that DCI improves metabolic and reproductive function in certain patients.

Our findings highlight once more that DCI supplementation causes a significant improvement in insulin sensitivity, delineated by a reduction in the HOMA index, along with a decrease in blood glucose and fasting insulin levels across both groups. Moreover, although the BMI was already significantly different at T0, the patients demonstrated significant body weight and BMI reductions in both the diet and DCI-supplemented groups. In contrast, our study, for the first time, questions the usefulness of high doses of this inositol to correct metabolic disturbances and the associated gynecological issues in a population different from healthy volunteers.

In detail, the administration of high-dose DCI resulted in alarming effects on hormonal profiles and menstrual cycle fluctuations. The control group exhibited a decline in total testosterone and androstenedione, likely due to the lowering of insulin levels and insulin-dependent production of ovarian androgens. In contrast, the treatment group experienced a noteworthy increase in the abovementioned androgens. Naturally, the FAI was also increased by treatment with high-dose DCI, whereas it was reduced in the diet-only group in agreement with the evidence in the literature (46). The increase in the FAI signifies that the women treated with high-dose DCI may have progressed toward a more hyperandrogenic phenotype, and this likely accounts for the menstrual irregularities observed in the treated group.

Due to the above-reported metabolic and hormonal concerns in the treatment group, the trial was stopped due to ethical considerations.

We speculate that these results are attributable to the endocrine role of DCI, which participates in steroidogenesis, downmodulating the conversion of androgens to estrogens via the aromatase enzyme. Indeed, as initially demonstrated by Sacchi using *in vitro* experiments (47) and later confirmed *in vivo* by Bevilacqua (48), DCI inhibits aromatase gene expression, promoting androgen accumulation at the expense of estrogens. In this regard, the literature agrees that DCI administration should be avoided in hyperandrogenic women with PCOS, who may experience a worsening of their symptoms.

Furthermore, a recent study (49) encouraged a more careful evaluation of doses, timing, and patient features in the case of a treatment with DCI, in order to achieve the desired therapeutic effect without exacerbating pathological conditions, such as PCOS-related hyperandrogenism (18).

Our study is not intended to confirm or explain the molecular mechanism underlying the clinical effects of DCI. Therefore, it is imperative that further research unravels the intricate interplay between DCI, insulin sensitivity, and hormonal regulation. Moreover, the importance of long-term studies cannot be overstated, as they are crucial for evaluating the sustainability of therapeutical effects and their broader clinical implications.

### Limitations

Among the limitations of this study, the primary one is the absence of a placebo in the control group, which may introduce potential bias; the relatively small sample size is the second.

## Conclusions

Overall, this study emphasizes how proper nutritional habits positively impact insulin sensitivity and excess weight. Undoubtedly, the joint use of combinational approaches may contribute to metabolic recovery; however, our study does not indicate any additional benefits of high-dose D-chiro-inositol supplementation compared to diet alone.

Moreover, our results indicate that it is crucial for clinicians to be aware of all known mechanisms of action of food supplements, in addition to dose- and time-related effects. It is hoped that this study may guide clinicians towards the prescription of tailored therapeutical strategies that address the patients’ individual needs.

## Data Availability

The data analyzed in this study is subject to the following licenses/restrictions: Data are available from the corresponding author upon reasonable request. Requests to access the datasets should be directed to sabrinabasciani@yahoo.it.

## References

[1] Johnson JD. On the causal relationships between hyperinsulinaemia, insulin resistance, obesity and dysglycaemia in type 2 diabetes. Diabetologia. (2021) 64:2138–46. doi: 10.1007/s00125-021-05505-4 34296322

[2] Deacon CF. Physiology and pharmacology of DPP-4 in glucose homeostasis and the treatment of type 2 diabetes. Front Endocrinol (Lausanne). (2019) 10:80. doi: 10.3389/fendo.2019.00080 30828317 PMC6384237

[3] Ginsberg HN. Insulin resistance and cardiovascular disease. J Clin Invest. (2000) 106:453–8. doi: 10.1172/jci10762 PMC380256 10953019

[4] Wild S Roglic G Green A Sicree R King H. Global prevalence of diabetes: estimates for the year 2000 and projections for 2030. Diabetes Care. (2004) 27:1047–53. doi: 10.2337/diacare.27.5.1047 15111519

[5] Li M Chi X Wang Y Setrerrahmane S Xie W Xu H. Trends in insulin resistance: insights into mechanisms and therapeutic strategy. Signal Transduct Target Ther. (2022) 7:216. doi: 10.1038/s41392-022-01073-0 35794109 PMC9259665

[6] Fahed M Abou Jaoudeh MG Merhi S Mosleh JMB Ghadieh R Al Hayek S. Evaluation of risk factors for insulin resistance: a cross sectional study among employees at a private university in Lebanon. BMC Endocr Disord. (2020) 20:85. doi: 10.1186/s12902-020-00558-9 32522257 PMC7288486

[7] Lippert K Kedenko L Antonielli L Kedenko I Gemeier C Leitner M. Gut microbiota dysbiosis associated with glucose metabolism disorders and the metabolic syndrome in older adults. Benef Microbes. (2017) 8:545–56. doi: 10.3920/bm2016.0184 28701081

[8] Baothman OA Zamzami MA Taher I Abubaker J Abu-Farha M. The role of Gut Microbiota in the development of obesity and Diabetes. Lipids Health Dis. (2016) 15:108. doi: 10.1186/s12944-016-0278-4 27317359 PMC4912704

[9] Shai I Schwarzfuchs D Henkin Y Shahar DR Witkow S Greenberg I. Weight loss with a low-carbohydrate, Mediterranean, or low-fat diet. N Engl J Med. (2008) 359:229–41. doi: 10.1056/NEJMoa0708681 18635428

[10] Greco M Chiefari E Montalcini T Accattato F Costanzo FS Pujia A. Early effects of a hypocaloric, Mediterranean diet on laboratory parameters in obese individuals. Mediators Inflammation. (2014) 2014:750860. doi: 10.1155/2014/750860 PMC3960747 24729662

[11] Menotti A Puddu PE. How the Seven Countries Study contributed to the definition and development of the Mediterranean diet concept: a 50-year journey. Nutr Metab Cardiovasc Dis. (2015) 25:245–52. doi: 10.1016/j.numecd.2014.12.001 25650160

[12] Mirabelli M Chiefari E Arcidiacono B Corigliano DM Brunetti FS Maggisano V. Mediterranean diet nutrients to turn the tide against insulin resistance and related diseases. Nutrients. (2020) 12(4):1066. doi: 10.3390/nu12041066 32290535 PMC7230471

[13] Unfer V Facchinetti F Orrù B Giordani B Nestler J. Myo-inositol effects in women with PCOS: a meta-analysis of randomized controlled trials. Endocr Connect. (2017) 6:647–58. doi: 10.1530/ec-17-0243 PMC5655679 29042448

[14] Greff D Juhász AE Váncsa S Váradi A Sipos Z Szinte J. Inositol is an effective and safe treatment in polycystic ovary syndrome: a systematic review and meta-analysis of randomized controlled trials. Reprod Biol Endocrinol. (2023) 21:10. doi: 10.1186/s12958-023-01055-z 36703143 PMC9878965

[15] Tabrizi R Ostadmohammadi V Lankarani KB Peymani P Akbari M Kolahdooz F. The effects of inositol supplementation on lipid profiles among patients with metabolic diseases: a systematic review and meta-analysis of randomized controlled trials. Lipids Health Dis. (2018) 17:123. doi: 10.1186/s12944-018-0779-4 29793496 PMC5968598

[16] Basciani S Nordio M Dinicola S Unfer V Gnessi L. Diet plus inositols, α-lactalbumin and gymnema sylvestre: the successful combo to restore body weight and metabolic profile in obese and dysmetabolic patients. Nutrients. (2023) 15(14):3142. doi: 10.3390/nu15143142 37513560 PMC10385591

[17] Genazzani AD Santagni S Rattighieri E Chierchia E Despini G Marini G. Modulatory role of D-chiro-inositol (DCI) on LH and insulin secretion in obese PCOS patients. Gynecol Endocrinol. (2014) 30:438–43. doi: 10.3109/09513590.2014.897321 24601829

[18] Dinicola S Unfer V Facchinetti F Soulage CO Greene ND Bizzarri M. Inositols: from established knowledge to novel approaches. Int J Mol Sci. (2021) 22(19):10575. doi: 10.3390/ijms221910575 34638926 PMC8508595

[19] Baillargeon JP Iuorno MJ Apridonidze T Nestler JE. Uncoupling between insulin and release of a D-chiro-inositol-containing inositolphosphoglycan mediator of insulin action in obese women With polycystic ovary syndrome. Metab Syndr Relat Disord. (2010) 8:127–36. doi: 10.1089/met.2009.0052 PMC3140116 20156067

[20] Facchinetti F Bizzarri M Benvenga S D'Anna R Lanzone A Soulage C. Results from the International Consensus Conference on Myo-inositol and d-chiro-inositol in Obstetrics and Gynecology: the link between metabolic syndrome and PCOS. Eur J Obstet Gynecol Reprod Biol. (2015) 195:72–6. doi: 10.1016/j.ejogrb.2015.09.024 26479434

[21] Bevilacqua A Bizzarri M. Physiological role and clinical utility of inositols in polycystic ovary syndrome. Best Pract Res Clin Obstet Gynaecol. (2016) 37:129–39. doi: 10.1016/j.bpobgyn.2016.03.007 27117028

[22] Kennington AS Hill CR Craig J Bogardus C Raz I Ortmeyer HK. Low urinary chiro-inositol excretion in non-insulin-dependent diabetes mellitus. N Engl J Med. (1990) 323:373–8. doi: 10.1056/nejm199008093230603 2370888

[23] Nordio M Basciani S Camajani E. The 40:1 myo-inositol/D-chiro-inositol plasma ratio is able to restore ovulation in PCOS patients: comparison with other ratios. Eur Rev Med Pharmacol Sci. (2019) 23:5512–21. doi: 10.26355/eurrev_201906_18223 31298405

[24] Obeid CA Gubbels JS Jaalouk D Kremers SPJ Oenema A. Adherence to the Mediterranean diet among adults in Mediterranean countries: a systematic literature review. Eur J Nutr. (2022) 61:3327–44. doi: 10.1007/s00394-022-02885-0 PMC9026058 35451614

[25] Cheang KI Baillargeon JP Essah PA Ostlund RE Jr. Apridonize T Islam L. Insulin-stimulated release of D-chiro-inositol-containing inositolphosphoglycan mediator correlates with insulin sensitivity in women with polycystic ovary syndrome. Metabolism. (2008) 57:1390–7. doi: 10.1016/j.metabol.2008.05.008 PMC2574418 18803944

[26] Nestler JE Jakubowicz DJ Reamer P Gunn RD Allan G. Ovulatory and metabolic effects of D-chiro-inositol in the polycystic ovary syndrome. N Engl J Med. (1999) 340:1314–20. doi: 10.1056/nejm199904293401703 10219066

[27] Yu HJ Ho M Liu X Yang J Chau PH Fong DYT. Association of weight status and the risks of diabetes in adults: a systematic review and meta-analysis of prospective cohort studies. Int J Obes (Lond). (2022) 46:1101–13. doi: 10.1038/s41366-022-01096-1 35197569

[28] De Lorenzo A Gratteri S Gualtieri P Cammarano A Bertucci P Di Renzo L. Why primary obesity is a disease? J Transl Med. (2019) 17:169. doi: 10.1186/s12967-019-1919-y 31118060 PMC6530037

[29] Jastreboff AM Kotz CM Kahan S Kelly AS Heymsfield SB. Obesity as a disease: the obesity society 2018 position statement. Obes (Silver Spring). (2019) 27:7–9. doi: 10.1002/oby.22378 30569641

[30] Stephenson J Smith CM Kearns B Haywood A Bissell P. The association between obesity and quality of life: a retrospective analysis of a large-scale population-based cohort study. BMC Public Health. (2021) 21:1990. doi: 10.1186/s12889-021-12009-8 34732156 PMC8567540

[31] Fernandes Silva L Vangipurapu J Laakso M. The "Common soil hypothesis" Revisited-risk factors for type 2 diabetes and cardiovascular disease. Metabolites. (2021) 11(10):691. doi: 10.3390/metabo11100691 34677406 PMC8540397

[32] Utzschneider KM Van de Lagemaat A Faulenbach MV Goedecke JH Carr DB Boyko EJ. Insulin resistance is the best predictor of the metabolic syndrome in subjects with a first-degree relative with type 2 diabetes. Obes (Silver Spring). (2010) 18:1781–7. doi: 10.1038/oby.2010.77 20379148

[33] Jeppesen J Hansen TW Rasmussen S Ibsen H Torp-Pedersen C Madsbad S. Insulin resistance, the metabolic syndrome, and risk of incident cardiovascular disease: a population-based study. J Am Coll Cardiol. (2007) 49:2112–9. doi: 10.1016/j.jacc.2007.01.088 17531661

[34] Saeedi P Petersohn I Salpea P Malanda B Karuranga S Unwin N. Global and regional diabetes prevalence estimates for 2019 and projections for 2030 and 2045: Results from the International Diabetes Federation Diabetes Atlas, 9(th) edition. Diabetes Res Clin Pract. (2019) 157:107843. doi: 10.1016/j.diabres.2019.107843 31518657

[35] Zeraattalab-Motlagh S Jayedi A Shab-Bidar S. Mediterranean dietary pattern and the risk of type 2 diabetes: a systematic review and dose-response meta-analysis of prospective cohort studies. Eur J Nutr. (2022) 61:1735–48. doi: 10.1007/s00394-021-02761-3 35001218

[36] Sarsangi P Salehi-Abargouei A Ebrahimpour-Koujan S Esmaillzadeh A. Association between adherence to the mediterranean diet and risk of type 2 diabetes: an updated systematic review and dose-response meta-analysis of prospective cohort studies. Adv Nutr. (2022) 13:1787–98. doi: 10.1093/advances/nmac046 PMC9526848 35472102

[37] Barrea L Vetrani C Verde L Frias-Toral E Ceriani F Cernea S. Comprehensive approach to medical nutrition therapy in patients with type 2 diabetes mellitus: from diet to bioactive compounds. Antioxidants (Basel). (2023) 12(4):904. doi: 10.3390/antiox12040904 37107279 PMC10135374

[38] D'Anneo A Lauricella M. Natural and synthetic compounds for management, prevention and treatment of obesity. Int J Mol Sci. (2022) 23(5):2890. doi: 10.3390/ijms23052890 35270032 PMC8910844

[39] Russell C Keshavamurthy S Saha S. Nutraceuticals in the management of cardiovascular risk factors: where is the evidence? Cardiovasc Hematol Disord Drug Targets. (2021) 21:150–61. doi: 10.2174/1871529x21666211201104124 34852755

[40] Unfer V Nestler JE Kamenov ZA Prapas N Facchinetti F. Effects of inositol(s) in women with PCOS: A systematic review of randomized controlled trials. Int J Endocrinol. (2016) 2016:1849162. doi: 10.1155/2016/1849162 27843451 PMC5097808

[41] Monastra G Gambioli R Unfer V Forte G Maymo-Masip E Comitato R. D-chiro-inositol and myo-inositol induce WAT/BAT trans-differentiation in two different human adipocyte models (SGBS and liSa-2). Int J Mol Sci. (2023) 24(8):7421. doi: 10.3390/ijms24087421 37108582 PMC10139407

[42] Formoso G Baldassarre MPA Ginestra F Carlucci MA Bucci I Consoli A. Inositol and antioxidant supplementation: Safety and efficacy in pregnancy. Diabetes Metab Res Rev. (2019) 35:e3154. doi: 10.1002/dmrr.3154 30889626 PMC6617769

[43] Iervolino M Lepore E Forte G Laganà AS Buzzaccarini G Unfer V. Natural molecules in the management of polycystic ovary syndrome (PCOS): an analytical review. Nutrients. (2021) 13(5):1677. doi: 10.3390/nu13051677 34063339 PMC8156462

[44] Zarezadeh M Dehghani A Faghfouri AH Radkhah N Naemi Kermanshahi M Hamedi Kalajahi F. Inositol supplementation and body mass index: A systematic review and meta-analysis of randomized clinical trials. Obes Sci Pract. (2022) 8:387–97. doi: 10.1002/osp4.569 PMC9159559 35664247

[45] Gambioli R Forte G Aragona C Bevilacqua A Bizzarri M Unfer V. The use of D-chiro-Inositol in clinical practice. Eur Rev Med Pharmacol Sci. (2021) 25:438–46. doi: 10.26355/eurrev_202101_24412 33506934

[46] Zapała B Marszalec P Piwowar M Chmura O Milewicz T. Reduction in the free androgen index in overweight women after sixty days of a low glycemic diet. Exp Clin Endocrinol Diabetes. (2024) 132:6–14. doi: 10.1055/a-2201-8618 38237611 PMC10796197

[47] Sacchi S Marinaro F Tondelli D Lui J Xella S Marsella T. Modulation of gonadotrophin induced steroidogenic enzymes in granulosa cells by d-chiroinositol. Reprod Biol Endocrinol. (2016) 14:52. doi: 10.1186/s12958-016-0189-2 27582109 PMC5006365

[48] Bevilacqua A Dragotto J Lucarelli M Di Emidio G Monastra G Tatone C. High doses of D-chiro-inositol alone induce a PCO-like syndrome and other alterations in mouse ovaries. Int J Mol Sci. (2021) 22(11):5691. doi: 10.3390/ijms22115691 34073634 PMC8198710

[49] Nordio M Bezerra Espinola MS Bilotta G Capoccia E Montanino Oliva M. Long-lasting therapies with high doses of D-chiro-inositol: the downside. J Clin Med. (2023) 12(1):390. doi: 10.3390/jcm12010390 36615188 PMC9821166

